# Enhancing the Impact of Implementation Strategies in Healthcare: A Research Agenda

**DOI:** 10.3389/fpubh.2019.00003

**Published:** 2019-01-22

**Authors:** Byron J. Powell, Maria E. Fernandez, Nathaniel J. Williams, Gregory A. Aarons, Rinad S. Beidas, Cara C. Lewis, Sheena M. McHugh, Bryan J. Weiner

**Affiliations:** ^1^Department of Health Policy and Management, Gillings School of Global Public Health, University of North Carolina at Chapel Hill, Chapel Hill, NC, United States; ^2^Cecil G. Sheps Center for Health Services Research, University of North Carolina at Chapel Hill, Chapel Hill, NC, United States; ^3^Frank Porter Graham Child Development Institute, University of North Carolina at Chapel Hill, Chapel Hill, NC, United States; ^4^Center for Health Promotion and Prevention Research, School of Public Health, University of Texas Health Science Center at Houston, Houston, TX, United States; ^5^School of Social Work, Boise State University, Boise, ID, United States; ^6^Department of Psychiatry, University of California, San Diego, La Jolla, CA, United States; ^7^Department of Psychiatry, Center for Mental Health, Perelman School of Medicine, University of Pennsylvania, Philadelphia, PA, United States; ^8^Department of Medical Ethics and Health Policy, Perelman School of Medicine, University of Pennsylvania, Philadelphia, PA, United States; ^9^Leonard Davis Institute of Health Economics, University of Pennsylvania, Philadelphia, PA, United States; ^10^MacColl Center for Healthcare Innovation, Kaiser Permanente Washington Health Research Institute, Seattle, WA, United States; ^11^School of Public Health, University College Cork, Cork, Ireland; ^12^Department of Global Health, Department of Health Services, University of Washington, Seattle, WA, United States

**Keywords:** implementation strategies, implementation science, designing and tailoring, mechanisms, effectiveness research, economic evaluation, reporting guidelines

## Abstract

The field of implementation science was developed to better understand the factors that facilitate or impede implementation and generate evidence for implementation strategies. In this article, we briefly review progress in implementation science, and suggest five priorities for enhancing the impact of implementation strategies. Specifically, we suggest the need to: (1) enhance methods for designing and tailoring implementation strategies; (2) specify and test mechanisms of change; (3) conduct more effectiveness research on discrete, multi-faceted, and tailored implementation strategies; (4) increase economic evaluations of implementation strategies; and (5) improve the tracking and reporting of implementation strategies. We believe that pursuing these priorities will advance implementation science by helping us to understand when, where, why, and how implementation strategies improve implementation effectiveness and subsequent health outcomes.

## Introduction

Nearly 20 years ago, Grol and Grimshaw (1) asserted that evidence-based practice must be complemented by evidence-based implementation. The past two decades have been marked by significant progress, as the field of implementation science has worked to develop a better understanding of implementation barriers and facilitators (i.e., determinants) and generate evidence for implementation strategies (2). In this article, we briefly review progress in implementation science and suggest five priorities for enhancing the impact of implementation strategies. We draw primarily upon the healthcare, behavioral health, and social services literature. While we hope the proposed priorities are applicable to studies conducted in a wide range of contexts, we welcome discussion regarding potential applications and enhancements for contexts outside of healthcare, such as community and public health settings (3) that often involve different types of stakeholders, interventions, and implementation strategies.

Implementation strategies are methods or techniques used to improve adoption, implementation, sustainment, and scale-up of interventions (4, 5). These strategies vary in complexity, from discrete or single component strategies (6, 7) such as computerized reminders (8) or audit and feedback (9) to multifaceted implementation strategies that combine two or more discrete strategies, some of which have been branded and tested using rigorous designs [e.g., (10, 11)]. Implementation strategies can target a range of stakeholders (12) and multilevel contextual factors across different phases of implementation (13–16). For example, strategies can address patient (17), provider (18), organizational (19), community (20, 21), policy and financing (22), or multilevel (23) factors.

Several taxonomies describe and organize the types of strategies available (6, 7, 24–26). Similarly, taxonomies of behavior change techniques (27) and methods (28) describe components of strategies at a more granular level. Both types of taxonomies promote a common language, inform implementation strategy development and evaluation by facilitating consideration of various “building blocks” or components of multifaceted and multilevel strategies, and improve the quality of reporting in research and practice.

The evidence base for implementation strategies is steadily developing. Initially, single-component, narrowly focused strategies that were effective in earlier studies were selected in subsequent studies despite differences between the clinical problems and contexts in which they were deployed (29). That approach was based on the assumption that strategies would be effective independent of the implementation problems being addressed (29). This “magic bullet” approach has led to limited success (30), prompting recognition that strategies should be selected or developed based upon a thorough understanding of context, including the causes of quality and implementation gaps, an assessment of implementation determinants, and an understanding of the mechanisms and processes needed to address them (29).

Evidence syntheses for discrete, multifaceted, and tailored implementation strategies have been conducted. The Cochrane Collaboration's Effective Practice and Organization of Care (EPOC) group has been a leader in this regard, with 132 systematic reviews of strategies such as educational meetings (31), audit and feedback (9), printed educational materials (32), and local opinion leaders (33). Grimshaw et al. (34) note that while median absolute effect sizes across implementation strategies are similar (see Table 1), the variation in observed effects within each strategy category suggests that effects may vary based upon whether or not they address determinants (barriers and facilitators). Indeed, determinants at multiple levels and phases may signal the need for multifaceted and tailored strategies that address key determinants (13).

**Table 1:** Evidence for common implementation strategies targeting professional behavior change.

**Meta-analyses**	**Number of studies/individuals**	**Effect sizes**
Printed educational materials (35)	14 RCTs and 31 ITS	Median absolute improvement of 2.0% (range 0% to 11%)
Educational meetings (31)	81 RCTs (involving more than 11,000 health professionals)	Median absolute improvement in care of 6.0% (interquartile range 1.8% to 15.3%)
Educational outreach (36)	69 RCTs (involving more than 15,000 health professionals)	Median absolute improvements in:-Prescribing behaviors [17 comparisons] of 4.8% (interquartile range 3.0–6.5%) -Other behaviors (e.g., providing screening tests; 17 comparisons) of 6.0% (interquartile range 3.6–16.0%)
Local opinion leaders (33)	18 RCTs (involving more than 296 hospitals and 318 primary care physicians)	Median absolute improvement of care of 12% across studies (interquartile range 6.0–14.5%)
Audit and feedback (9)	140 RCTs	Median absolute improvement of 4.3% (interquartile range 0.5–16%)
Computerized reminders (8)	28 RCTs	Median absolute improvement of care 4.2% (interquartile range 0.8–18.8%)
Tailored implementation strategies (37)	32 RCTs	Meta-regression using 15 randomized trials. Pooled odds ratio of 1.56 (95% CI, 1.27–1.93, *p* < 0.001)

*Table updated from Grimshaw et al. (34), and draws upon Cochrane Reviews from the Effective Practice and Organization of Care (EPOC) group (38)*.

While the use of multifaceted and tailored implementation strategies is intuitive and has considerable face validity (29), the evidence regarding their superiority to single-component strategies has been mixed (37, 39, 40). A review of 25 systematic reviews (39) found “no compelling evidence that multifaceted interventions are more effective than single-component interventions” (p. 20). Grimshaw et al. (34) provide one possible explanation, emphasizing that the general lack of an *a priori* rationale for the selection of components (i.e., discrete strategies) in multifaceted implementation strategies makes it difficult to determine how these decisions were made. They may have been selected thoughtfully to address prospectively identified determinants through theoretically- or empirically-derived change mechanisms, or they may simply be the manifestation of a “kitchen sink” approach. Wensing et al. (41) offer a complementary perspective, noting that definitions of discrete and multifaceted strategies are problematic. A discrete strategy such as outreach visits may include instruction, motivation, planning of improvement, and technical assistance; thus, it may not be accurate to characterize it as a single-component strategy. Conversely, a multifaceted strategy including educational workshops, educational materials, and webinars may only address provider knowledge and fail to address other important implementation barriers. They propose that multifaceted strategies that truly target multiple relevant implementation determinants could be more effective than single-component strategies (41).

A systematic review of 32 studies testing strategies tailored to address determinants concluded that tailored approaches to implementation were more effective than no strategy or a strategy not tailored to determinants; however, the methods used to identify and prioritize determinants and select implementation strategies were not often well-described and no specific method has been proven superior (37). The lack of systematic methods to guide this process is problematic, as evidenced by a review of 20 studies that found that implementation strategies were often poorly conceived, with mismatches between strategies and determinants (e.g., barriers were identified at the team or organizational level, but strategies were not focused on structures and processes at those levels) (42). A multi-national program of research was undertaken to improve the methods of tailoring implementation strategies (43), but tailored strategies had little impact on primary and secondary outcomes (40). Questions remain about the best methods to develop tailored implementation strategies.

Five priorities need to be addressed to increase the public health impact of implementation strategies: (1) enhance methods for designing and tailoring; (2) specify and test mechanisms of change; (3) conduct more effectiveness research on discrete, multifaceted, and tailored strategies; (4) increase economic evaluations; and (5) improve tracking and reporting. Table 2 provides examples of studies that have pursued each priority with rigor.

**Table 2:** Five priorities for research on implementation strategies.

**Priority**	**Example(s)**
1. Enhance methods for designing and tailoring implementation strategies	Highfield et al. (44) used Intervention Mapping to systematically design an implementation strategy for an evidence-based mammography intervention.
2. Specify and test mechanisms of change	Williams et al. (45) assessed mechanisms of change for the ARC organizational strategy using multilevel mediation analysis, and found that ARC increases adoption of evidence-based practices by creating proficient organizational cultures that increase clinicians' intentions to adopt evidence-based practices.
3. Conduct more effectiveness research on discrete, multi-faceted, and tailored implementation strategies	*Discrete*: Gude et al. (46) detail an approach to identifying how a discrete strategy (electronic audit and feedback) works and how it might be optimized. *Multi-faceted*: Kilbourne et al. (47) are using a sequential multiple assignment randomized trial (SMART) design to build an adaptive implementation strategy, demonstrating how trials may be useful in determining the appropriate intensity and sequencing of components in multifaceted strategies. *Tailored*: The Tailored Implementation for Chronic Diseases project provides several examples of trials of tailored implementation strategies and corresponding process evaluations, which are documented in an article collection (48). Wensing details opportunities for improving tailored implementation strategies (40).
4. Increase economic evaluations of implementation strategies	Hoomans and Severens (49) detail how economic analyses apply to implementation science, and provide numerous examples in which cost-effectiveness analyses can facilitate decision-making related to implementation strategies.
5. Improve tracking and reporting of implementation strategies	*Tracking*: Bunger et al. (50) and Boyd et al. (51) have demonstrated how implementation strategy reporting guidelines (4) can be adapted and applied to prospectively track strategy use over the course of an implementation effort. *Reporting*: Bunger et al. (52) used the Proctor et al. (4) guidelines to retrospectively report key components of a learning collaborative intended to increase the use of Trauma-Focused Cognitive Behavioral Therapy.

## Enhance Methods for Designing and Tailoring Implementation Strategies

Implementation strategies are too often designed in an unsystematic manner and fail to address key contextual determinants (13–16). Stakeholders may rely upon inertia (i.e., “we've always done things this way”), one size fits all approaches, or utilize what Martin Eccles has called the ISLAGIATT principle (i.e., “it seemed like a good idea at the time”) (53). Consequently, strategies are not always well-matched to the contexts in which they are deployed, including the interventions to be implemented, settings, stakeholder preferences, and implementation determinants (37, 42, 54). More rational, systematic approaches to identify and prioritize barriers and link strategies to overcome them are needed (37, 42, 55–57). A number of methods have been suggested. Colquhoun and colleagues (56) found 15 articles with replicable methods for designing strategies to change healthcare professionals' behavior, and Powell et al. (55) proposed Intervention Mapping (58), concept mapping (59), conjoint analysis (60), and system dynamics modeling (61) as methods to aid the design, selection, and tailoring of strategies. These methods share common steps (identification of barriers, linking barriers to strategy component selection, use of theory, and user engagement), and have potential to make the process of designing and tailoring implementation strategies more rigorous (55, 56). For example, Intervention Mapping is step-by-step approach to developing implementation strategies using a detailed and participatory needs assessment and the identification of implementers, implementation behaviors, determinants, and ultimately, behavior change methods and implementation strategies that influence determinants of implementation behaviors. Some work has been done to compare different methods for assessing determinants (62); however, several questions remain. How can determinants be accurately and efficiently assessed (ideally leveraging implementation frameworks)? Can perceived and actual determinants be differentiated? What are the best methods for prioritizing determinants that need to be proactively addressed? When should determinant assessment take place given that new challenges are likely to emerge during the course of implementation? Who should be involved in this process? Each of those questions has resource implications. Similarly, questions remain about efficiently linking prioritized determinants to effective and pragmatic implementation strategies. How can causal theory be leveraged or developed to guide the selection of implementation strategies? Can pragmatic tools be developed to systematically link strategies to determinants? Approaches to designing and tailoring implementation strategies should be tested to determine whether they improve implementation and clinical outcomes (55, 56). Given that clinical problems, clinical and public health interventions, settings, individuals, and contextual factors are highly heterogeneous, there is much to gain from developing generalizable processes for designing and tailoring strategies.

## Specify and Test Mechanisms of Change

Studies of implementation strategies should increasingly focus on establishing the processes and mechanisms by which strategies exert their effects rather than simply establishing whether or not they were effective (29, 63, 64). The National Institutes of Health (64) provides this guidance:

Wherever possible, studies of dissemination or implementation strategies should build knowledge both on the overall effectiveness of the strategies, as well as “how and why” they work. Data on mechanisms of action, moderators, and mediators of dissemination and implementation strategies will greatly aid decision-making on which strategies work for which interventions, in which settings, and for which populations.

Unfortunately, it is not common that mechanisms are even mentioned, much less tested (63, 65, 66). Williams (63) emphasizes the need for trials that test a wider range of multilevel mediators of implementation strategies, stronger theoretical links between strategies and hypothesized mediators, improved design and analysis of multilevel mediation models in randomized trials, and an increasing focus on identifying implementation strategies and behavior change techniques that contribute most to improvement. Developing a more nuanced understanding of mechanisms will require researchers to thoroughly assess the context of implementation *and* describe causal pathways by which strategies exert their effects, moving beyond a broad identification of determinants and articulating mediators, moderators, preconditions, and proximal and distal outcomes (67). Examples of this type of approach and guidance for their development can be found in Lewis et al. (67), Weiner et al. (23), Bartholomew et al. (58), and Highfield et al. (44). Additionally, drawing more heavily upon theory (66, 68, 69), using research designs that maximize ability to make causal inferences (70, 71), leveraging methods that capture and reflect the complexity of implementation such as systems science (61, 72, 73) and mixed methods (74–76) approaches, and adhering to methods standards for studies of complex interventions (77) will help to sharpen our understanding of how implementation strategies engage hypothesized mechanisms. Work to link implementation strategies and behavior change techniques to hypothesized mechanisms is underway (67, 78), which promises to improve our understanding of how, when, where, and why implementation strategies are effective.

## Conduct More Effectiveness Research on Discrete, Multi-faceted, and Tailored Implementation Strategies

There is a need for more and better effectiveness research on discrete, multifaceted, and tailored implementation strategies using a wider range of innovative designs (70, 79–82). First, while a number of discrete implementation strategies have been described (6, 7, 24, 25) and tested (38), there are gaps in our understanding about how to optimize these strategies. There are over 140 randomized trials of audit and feedback, but Ivers et al. (83) conclude that there is much to learn about when it will work best and why, and how to design reliable and effective audit and feedback strategies across different settings and providers. Audit and feedback is an example of how complex implementation strategies can be. The ICeBERG group (69) pointed to the fact that even varying five modifiable elements of audit and feedback (content, intensity, method of delivery, duration, and context) produces 288 potential combinations. These variations matter (84), and there is a need for tests of audit and feedback and other discrete implementation strategies that include clearly described components that are theoretically and empirically derived, and well-operationalized. The results of these studies could inform the use of discrete strategies and their inclusion in multifaceted strategies.

Second, there is a need for trials that give insight into the sequencing of multifaceted strategies and what to do if the first strategy fails (39). These strategies could be compared to discrete/single-component implementation strategies or multifaceted strategies of varying complexity and intensity with well-defined components that are theoretically aligned with implementation determinants. These strategies could be tested using MOST, SMART, or other variants of factorial designs that can evaluate the relative impact of various components of multifaceted strategies and inform their sequencing (70, 85).

Finally, tests of strategies that are prospectively tailored to different implementation contexts to address specific implementers, implementation behaviors, or determinants are needed (37). This work could involve comparisons between tailored and non-tailored multifaceted implementation strategies (86), as well as tests of established and innovative methods that could inform the identification, selection, and tailoring of implementation strategies (55, 56).

## Increase Economic Evaluations of Implementation Strategies

Few studies include economic evaluations of implementation strategies (87, 88). For example, in a systematic review of 235 implementation studies, only 10% provided information about implementation costs (87). The dearth of economic evaluations severely limits our ability to understand which strategies might be feasible for different contexts, as some decision makers might underestimate the resources required to implement and sustain EBPs, while others might over-estimate them and preemptively limit themselves from implementing EBPs that could benefit their communities (89). Incorporating economic analyses into studies of implementation strategies would provide decision makers more complete information to guide strategy selection, and would encourage researchers to be more judicious and pragmatic in their design and selection of implementation strategies, narrowing attention to strategies and mechanisms hypothesized to be most essential. If methods for designing and tailoring strategies can be improved such that complex multifaceted strategies are proven superior to single-component or less complex multifaceted strategies (39) and tailored strategies are proven superior to more standard multifaceted strategies (37, 40, 43, 55), economic evaluations will be instrumental in demonstrating whether improvements in implementation are worth added costs. Practical tools for integrating economic evaluations within implementation studies have been developed, such as the Costs of Implementing New Strategies (COINS) method (89) which was developed to address the need for standardized methods for analyzing cost data in implementation research that extend beyond the cost of the clinical intervention itself (90). For example, the original COINS study presented a head-to-head trial of two implementation approaches; although one approach was significantly more costly, the implementation outcomes achieved were superior enough to warrant the additional resources (91). Increasing the number and relevance of economic evaluations will require the development of a common framework that promotes comparability across studies (88).

## Improve Tracking and Reporting of Implementation Strategies

Developing a robust evidence base for implementation strategies will require that their use be contemporaneously tracked and that they be reported in the literature with sufficient detail (92). It is often difficult to ascertain which implementation strategies were used and how they might be replicated. Part of the challenge is the iterative nature of implementation. Even if strategies are meticulously described in a study protocol or trial registry, it is often unrealistic to expect that they will not need to be altered as determinants emerge across implementation phases (13, 93, 94). These changes are likely to occur within and between implementing sites in research studies and applied efforts (50, 51), and without rigorous methods for tracking implementation strategy use, efforts to understand what strategies were used and whether or not they were effective are stymied. Even when strategies are reported in study protocols or empirical articles, there are numerous problems with their description, including inconsistent labeling; lack of operational definitions; poor description and absence of manuals to guide their use; and lack of a clear theoretical, empirical, or pragmatic justification for how the strategies were developed and applied (4). Poor reporting clouds the interpretation of results, precludes replication in research and practice, and limits our ability to synthesize findings across studies (4, 92). Findings from systematic reviews illustrate this problem. For example, Nadeem et al. (95) review of learning collaboratives concluded that, “reporting on specific components of the collaborative was imprecise across articles, rendering it impossible to identify active quality improvement collaborative ingredients linked to improved care.”

A number of reporting guidelines could be leveraged to improve descriptions of strategies (4, 96–100). Proctor et al. (4) recommend that researchers name and define strategies in ways that are consistent with the published literature, and carefully operationalize the strategy by specifying: (1) *actor(s)*, (2) *action(s)*, (3) *action target(s)*, (4) *temporality*, (5) *dose*, (6) *implementation outcomes affected*, and (7) theoretical, empirical, or pragmatic *justification*. Specifying strategies in this way has the potential to increase our understanding of not only which strategies are most effective, but more importantly, the processes and mechanisms by which they exert their effects (29, 67). Additional options that provide structured reporting recommendations include the Workgroup for Intervention Development and Evaluation Research (WIDER) recommendations (99, 100), the Simplified Framework (96) and its extension [AIMD; (97)], and the Template for Intervention Description and Replication (TIDieR) checklist (98). Though not specific to the reporting of implementation strategies, the Standards for Reporting Implementation Studies (101) and Neta et al. (102) reporting framework emphasizes how critical it is to report on the multilevel context of implementation. The use of *any* of the existing guidelines would enhance the clarity of strategy description. We believe that developing approaches to tracking implementation strategies (50, 51), and assessing the extent to which they are pragmatic (e.g., acceptable, compatible, easy, and useful) for both research and applied efforts is a high priority. Further, efficient ways of linking empirical studies with study protocols to gauge the degree to which strategies have been adapted or tailored over the course of an implementation effort would be helpful. Failing to improve the quality of reporting will negate other advances in this area by hindering replication.

## Conclusion

Implementation science has advanced considerably, yielding a more robust understanding of implementation strategies. Several resources can inform the use of implementation strategies, including established taxonomies of implementation strategies (6, 7, 24, 25) and behavior change techniques (27, 28), repositories of systematic reviews (38, 103, 104), methods for selecting and tailoring implementation strategies (40, 55, 56), and reporting guidelines that promote replicability (4, 98–100). Nevertheless, questions remain and further effectiveness research and methodological development are needed to ensure that evidence is effectively translated into public health impact. Advancing these priorities will lead to a better understanding of when, where, why, and how implementation strategies exert their effects (29, 63).

## Author Contributions

BP conceptualized the paper and wrote the first draft of the manuscript. All other authors contributed to the writing and approved the final manuscript.

### Conflict of Interest Statement

The authors declare that the research was conducted in the absence of any commercial or financial relationships that could be construed as a potential conflict of interest.
